# Caspase-dependent and -independent suppression of apoptosis by monoHER in Doxorubicin treated cells

**DOI:** 10.1038/sj.bjc.6603598

**Published:** 2007-02-06

**Authors:** A M E Bruynzeel, M A Abou El Hassan, E Torun, A Bast, W J F van der Vijgh, F A E Kruyt

**Affiliations:** 1 Department of Medical Oncology, VU University Medical Center, 1081 HV, Amsterdam, The Netherlands; 2 Cancer Biology Department, National Cancer Institute, Cairo University, Cairo, Egypt; 3 Department of Pharmacology and Toxicology, Faculty of Medicine, University of Maastricht, 6200 MD, Maastricht, The Netherlands

**Keywords:** monoHER, doxorubicin, apoptosis, caspases, (non) tumour cells

## Abstract

Doxorubicin (DOX) is an antitumour agent for different types of cancer, but the dose-related cardiotoxicity limits its clinical use. To prevent this side effect we have developed the flavonoid monohydroxyethylrutoside (monoHER), a promising protective agent, which did not interfere with the antitumour activity of DOX. To obtain more insight in the mechanism underlying the selective protective effects of monoHER, we investigated whether monoHER (1 mM) affects DOX-induced apoptosis in neonatal rat cardiac myocytes (NeRCaMs), human endothelial cells (HUVECs) and the ovarian cancer cell lines A2780 and OVCAR-3. DOX-induced cell death was effectively reduced by monoHER in heart, endothelial and A2780 cells. OVCAR-3 cells were highly resistant to DOX-induced apoptosis. Experiments with the caspase-inhibitor zVAD-fmk showed that DOX-induced apoptosis was caspase-dependent in HUVECs and A2780 cells, whereas caspase-independent mechanisms seem to be important in NeRCaMs. MonoHER suppressed DOX-dependent activation of the mitochondrial apoptotic pathway in normal and A2780 cells as illustrated by p53 accumulation and activation of caspase-9 and -3 cleavage. Thus, monoHER acts by suppressing the activation of molecular mechanisms that mediate either caspase-dependent or -independent cell death. In light of the current work and our previous studies, the use of clinically achievable concentrations of monoHER has no influence on the antitumour activity of DOX whereas higher concentrations as used in the present study could influence the antitumour activity of DOX.

Doxorubicin (DOX) is highly effective against various types of cancers, including leukaemias, breast and ovarian cancer. Doxorubicin (DOX) induces pleiotropic cytotoxic effects of which DNA intercalation and topoisomerase II inhibition have been proposed to play an important role in its mechanism of action, causing growth arrest and the subsequent activation of apoptosis (Capranico *et al*, 1990; Gewirtz, 1991; Binaschi *et al*, 1997). The contribution of DOX-induced reactive oxygen species (ROS) to its antitumour activity is still a matter of debate (Keizer *et al*, 1991; Shacter *et al*, 2000; Gouaze *et al*, 2001; Suresh *et al*, 2003).

A major limitation of the use of DOX in the clinic is its cumulative dose-related cardiotoxicity, which ultimately can lead to severe and irreversible forms of cardiomyopathy (Lefrak *et al*, 1973; Singal and Iliskovic, 1998; Hrdina *et al*, 2000). Doxorubicin (DOX)-induced ROS have been shown as the primary cause of the heart damaging effect of the drug (Horenstein *et al*, 2000; Xu *et al*, 2001). In addition, several studies suggested that DOX-induced apoptosis in endothelial cells and cardiomyocytes contributes to the development of DOX-related cardiotoxicity (Childs *et al*, 2002; Wu *et al*, 2002; Kluza *et al*, 2004; Spallarossa *et al*, 2004). Various strategies have been developed to protect against DOX-induced cardiotoxic effects. The majority of these studies concern either metal ion chelators or antioxidants that yielded moderate protective effects (Swain *et al*, 1997; Kotamraju *et al*, 2000; Liu *et al*, 2002; Yamanaka *et al*, 2003; Oliveira *et al*, 2004).

MonoHER — a semisynthetic flavonoid that was developed in our laboratory (Willems *et al*, 2006) and presently in a phase II clinical trial is both a radical scavenging and a metal ion chelating agent (Haenen *et al*, 1993; van Acker *et al*, 1993). In our studies the scavenging activity of monoHER was measured by determining the inhibition of ferricytochrome c reduction and the inhibition of oxygen consumption, showing an inhibition of 91 and 70%, respectively (van Acker *et al*, 1993). Because of these favourable properties, monoHER was tested as a protectant against DOX-induced cardiac damage in *in vivo* models and appeared a potent protector against DOX-induced cardiotoxicity without influencing its antitumour effects (van Acker *et al*, 1997, 2000).

The present study was initiated to determine possible differences in the effects of monoHER against DOX-induced apoptosis in cardiac myocytes and vascular endothelial cells in comparison to ovarian cancer cell lines. The protective effects of monoHER on cell cycle progression and molecular determinants of mitochondrial-dependent apoptosis were also studied. The clinical relevance of our results regarding the use of protectors such as monoHER in combination with chemotherapy is discussed.

## MATERIALS AND METHODS

### Chemicals

7-Monohydroxyethylrutoside (monoHER) was kindly provided by Novartis Consumer Health (Nyon, Switzerland). Doxorubicin HCl was purchased from Pharmacia Upjohn BV (Woerden, the Netherlands). The following reagents were used in this study: bovine serum albumin, trypsin, 3-(4,5-dimethylthiazol-2-yl)-2,5-diphenyltetrazolium bromide (MTT) and sodium vanadate (NaVO_3_) (all from Sigma-Aldrich Chemie (Zwijndrecht, the Netherlands)); human serum (Central Laboratory for Blood Transfusion, Amsterdam, the Netherlands); M199 medium and Hanks Balanced salt solution (without calcium) (Invitrogen, Breda, the Netherlands); Dulbecco's modified Eagle's medium (DMEM) and HEPES (Cambrex Bio Science, New Jersey, USA); fetal calf serum (FCS) (Greiner Bio-One B.V., Alphen a/d Rijn, the Netherlands); Na-penicillin-G (Yamanouchi Pharma B.V., the Netherlands); streptomycin (Fisiopharma, Milano, Italy); L-glutamine (ICN Biochemicals, Cleveland, Ohio, USA); horse serum (Life Technologies NV, Merelbeke, Belgium); glycerol (J.T. Baker, Europe, Deventer, the Netherlands); protease inhibitor tablets (Roche Diagnostics Netherland BV, Almere, the Netherlands); Benzyloxycarbonyl-Val-Ala-Asp-fluoromethyl keton (z-VAD) (Enzyme Systems, Livermore, CA, USA); Endothelial cell growth factor (ECGF) was extracted from bovine hypothalamus as described previously (Maciag *et al*, 1979).

### Cell culture

All cells were maintained at 37°C in humidified air containing 5% CO_2_. A2780 and OVCAR-3 cells were grown in DMEM with 10% FCS and 20 mM HEPES. Human endothelial cells (HUVECs) were prepared as described previously by Verheul *et al* (2000). In brief, the endothelial cells were cultured in gelatin-coated (1%) tissue culture plates with culture medium consisting of M199 supplemented with 10% human serum, 10% FCS, 5 U ml^−1^ heparin, 200 IE ml^−1^ penicillin and 200 *μ*g ml^−1^ streptomycin, 0.29 mg ml^−1^ L-glutamine and 50 *μ*g ml^−1^ ECGF. Cells were grown to confluence at 37°C in 5% CO_2_. Endothelial cells after three passages (P3) were used during the whole study. Neonatal rat cardiac myocytes (NeRCaMs) were isolated as described previously by (Abou El Hassan *et al*, 2003a). The cardiac cells were divided over tissue culture plates with a cell density of 75 × 10^3^ cells cm^−2^. After plating, the cells were incubated with medium consisting of equal portions of DMEM and Ham's F-10 supplemented with 100 U ml^−1^ penicillin, 100 *μ*g ml^−1^ streptomycin and 5% horse serum. The cultures contained at least 90% of synchronously beating NeRCaMs. After 24 h the medium was refreshed and 48 h later the NeRCaM cultures were ready for use in the experiments.

### Flow cytometric analysis of PI-stained cells

Cardiac myocytes, endothelial, A2780 and OVCAR-3 cells were treated with different concentrations of DOX (0.1–10 *μ*M) alone or in the presence of 1 mM monoHER for 48 h, respectively. Apoptotic cell fraction (sub-G1) in PI-stained cells was measured by flow cytometry as described previously (Swain *et al*, 1997; Suresh *et al*, 2003). In brief, after trypsinisation the cells were resuspended in PI staining solution (50 *μ*M ml^−1^ PI, 0.1% sodium citrate, 0.1% TritonX-100, 0.1 mg ml^−1^ Rnase in PBS). The cells were incubated for at least 30 min at 4°C in the dark before analysis by flow cytometry using a FACScan (Becton, Dickinson and Company, NJ, USA). When indicated cells were pretreated for 1 h with 50 *μ*M of the broad caspase inhibitor z-VAD-fmk before DOX exposure or co-treated with monoHER (1 mM).

### Western blot analysis

Cells were treated with DOX with or without 1 mM MonoHER for 24 and 48 h at 37°C. The cells were trypsinised and lysed for 20 min on ice in lysis buffer consisting of 20 mM HEPES/KOH (pH 7.4), 50 mM *β*-glycerophosphate, 50 mM KCl, 0.2 mM EDTA, 1% (w/v) Triton X-100, and 10% (w/v) glycerol, supplemented with protease inhibitors and 1 mM NaVO_3_. Protein concentrations were determined using the Bio-Rad assay (Bio-Rad Laboratories, Richmond, CA, USA) with BSA as a standard using a spectrophotometer. Equal amounts of protein (15 *μ*g) were loaded and electrophoresed on 7–12% SDS–polyacrylamide gels and transferred into polyvinylidene difluoride membranes (Amersham, Braunschweig, Germany). Subsequently, membranes were blocked with 5% non-fat dry milk for 1 h at room temperature and incubated at 4°C overnight with the indicated primary antibodies followed by a 1 h incubation at room temperature with a secondary antibody, either horseradish peroxidase-conjugated goat anti-mouse or goat anti-rabbit antibodies (1 : 2000). Protein loading equivalence was assessed by the expression of *β*-actin (1 : 10 000). Proteins were visualised by enhanced chemiluminescence (ECL kit, Amersham, Braunschweig, Germany). Primary antibodies used were against p53 (human specific, DAKO, Glostrup, Denmark), poly(ADP-ribose) polymerase (PARP) (Roche Diagnostics Netherland BV, Almere, the Netherlands), caspase-9 (human specific or mouse mAb), p53 and PARP (rat specific, all from Cell Signaling Technology Inc., Beverly, MA, USA), Bax and caspase-3 (BD Transduction Laboratories NJ, USA) and *β*-actin (Sigma-Aldrich Chemie, Zwijndrecht, the Netherlands).

## RESULTS

### Effect of monoHER on DOX-induced apoptosis

**Figure 1:**
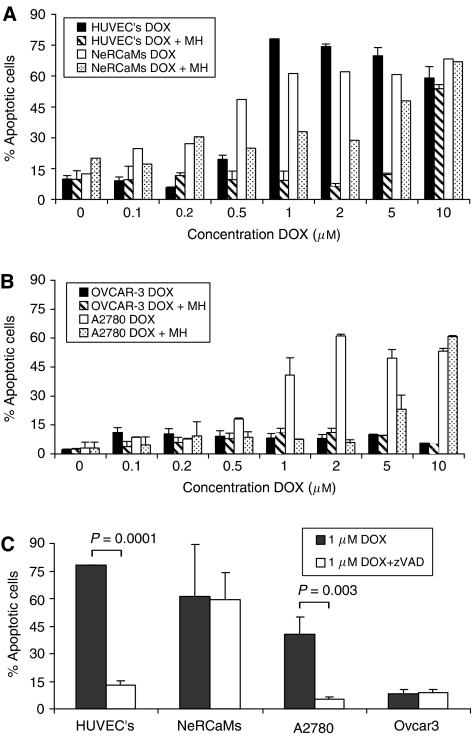
The effect of monoHER (1 mM) on DOX-induced apoptosis in cardiomyocytes (NeRCaMs), endothelial cells (HUVECs) (**A**) and ovarian tumour cells A2780 and OVCAR-3 (**B**) after 48 h of incubation. The bars indicate the levels of the sub-G1 cell fractions of apoptotic cells after PI staining. The effect of 50 *μ*M zVAD-fmk (a broad-spectrum caspase inhibitor) on DOX (1 *μ*M, 48 h)-induced cell death was also investigated (**C**). Results are presented as the mean (±s.d.) of three independent experiments except for the NeRCaMs in Figure 1A, where results of two experiments are shown.

Normal cells were more sensitive to DOX-induced apoptosis than the selected tumour cell lines. The IC_50_ values were 0.75, 0.5 and 1.5 *μ*M for HUVECS, NeRCaMs and A2780 cells, respectively. However, OVCAR-3 was highly resistant with a maximum of only 10–12% cells in sub-G1 phase at 10 *μ*M (Figure 1 and Table 1). HUVECs were relatively resistant to low concentrations of DOX and reached a plateau of apoptotic cell fraction at a concentration of 1 *μ*M. NeRCaMs displayed a gradual dose-dependent increase of apoptotic cells at low DOX concentrations reaching a plateau of 60% killing at 1 *μ*M DOX. A2780 cells also showed a dose-dependent increase in apoptotic cells, reaching a plateau of about 50% at DOX concentrations ⩾1 *μ*M. The ratios of the IC_50_ values shown in Table 1 indicate that 1 mM MonoHER protected 13- and 15-fold against DOX-induced apoptosis in HUVECs and NeRCaMs, respectively. A2780 cells were only protected with a factor 5.3, whereas OVCAR-3 did not show any protection by monoHER.

### Doxorubicin-induced caspase activation

The differences found in sensitivity to the apoptotic effects of DOX in the examined cell panel prompted us to investigate the role of caspases in mediating cell death. To that purpose, HUVECs, NeRCaMs, A2780 and OVCAR-3 cells were treated with 1 *μ*M DOX with or without the addition of the broad-spectrum caspase inhibitor z-VAD-fmk (Figure 1C). A strong suppression (leaving only 15% apoptotic cells) was observed in HUVECs whereas no significant protection was observed in NeRCaMs. In tumour cells, z-VAD-fmk almost completely protected against the moderate apoptotic effects of DOX in A2780 cells (only 10% apoptotic cells left). Z-VAD-fmk exhibited no significant protection against DOX-induced apoptotic effects in OVCAR-3; however the interpretation of this finding is difficult in light of the strong apoptosis resistance found in these cells. Taken together, these results indicate that DOX-induced apoptosis in HUVECs and A2780 cells was principally mediated by caspases, whereas NeRCaMs and OVCAR-3 cells displayed less or hardly any contribution of caspases to DOX toxicity. In addition, the effect of a higher dose of DOX (10 *μ*M) with or without the addition of z-VAD-fmk was examined inducing a more robust cell death than 1 *μ*M DOX. Under these conditions, z-VAD-fmk showed comparable results as obtained at 1 *μ*M DOX (results not shown).

### Cell cycle effects of DOX treatment

**Figure 2:**
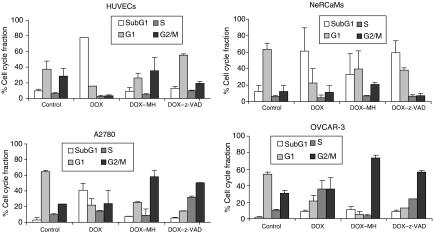
Effect of 1 mM monoHER and 50 *μ*M zVAD-fmk on cell cycle progression in cardiomyocytes, endothelial cells and ovarian cancer cells treated with 1 *μ*M DOX for 48 h. The percentage of cells in the different cell cycle fractions was determined by flow cytometry.

The effect of the combined treatment of DOX (1 *μ*M, 48 h) with monoHER or z-VAD-fmk on cell cycle progression in the panel of non-tumour and tumour cells was determined by FACS analysis of PI-stained cells (Figure 2). Doxorubicin (DOX) alone induced apoptosis as illustrated by the accumulation of cells in sub-G1. HUVECs were most sensitive to DOX and monoHER-dependent suppression of DOX-induced sub-G1 accumulation was clearly accompanied by G2/M arrest. The cell cycle profile obtained after caspase inhibition displayed accumulation of HUVECs in all cell cycle phases with a distribution resembling that of untreated control cells. In NeRCaMs co-exposure with monoHER protected against apoptosis, and a small increase in G2/M accumulation was observed. A2780 tumour cells showed strong accumulation in G2/M upon apoptosis suppression by co-treatment with monoHER; z-VAD-fmk also led to G2/M accumulation and strong S-phase arrest. In apoptosis-resistant OVCAR-3 cells DOX mediated an S-phase arrest that was strongly prevented by monoHER, leading to a large accumulation in G2/M. Co-treatment with z-VAD-fmk resulted in accumulation of cells in the G2/M phase.

Taken together, it appears that monoHER delays DOX-treated cells in the G2/M phase, particularly in the examined tumour cells. Caspase inhibition by z-VAD-fmk was most notable in HUVECs where DOX apoptotic effects seem to be merely caspase-dependent, whereas other cell types showed partial inhibition or in case of OVCAR-3 almost no effect at all. Although both monoHER and z-VAD-fmk had overlapping effects on the cell cycle distribution of DOX-treated cells, mainly G2/M accumulation, z-VAD-fmk resulted also in the delay of the tumour cells in the S phase, and in the G1 phase in case of HUVECs or NeRCaMs. Because monoHER can suppress DOX cytotoxicity in all these cells it apparently suppresses both caspase-dependent and -independent forms of cell death.

### Effect of monoHER on DOX-dependent activation of p53, Bax and caspases

To obtain more insight into the molecular mechanism underlying the apoptosis-inducing effect of DOX and the apoptosis suppressing effects of monoHER, the expression of several regulators and executors of apoptosis was studied in the panel of cells by Western blotting. To that purpose, cells were exposed to the corresponding IC_50_ concentrations of DOX in the presence or absence of monoHER for 24 and 48 h.

**Figure 3:**
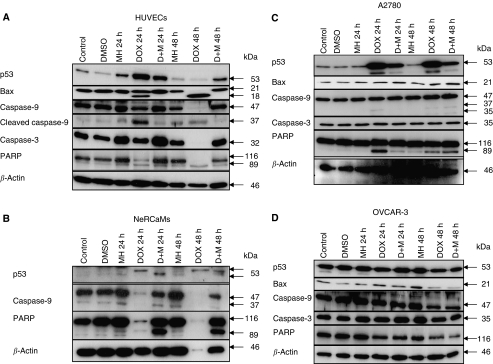
Effect of monoHER on DOX-induced p53, Bax and caspase activation. The expression of the indicated proteins was examined by Western blotting in HUVECs (**A**), NeRCaMs (**B**), and in A2780 (**C**) and OVCAR-3 tumour cells (**D**) after 24 and 48 h of DOX exposure at their IC_50_. The molecular weight of the bands is indicated and *β*-actin was included as a control for protein loading.

In HUVECs (Figure 3A), DOX treatment resulted in an accumulation of p53 at 24 h exposure which decreased at 48 post-treatment, most probably caused by the strong activation of apoptosis resulting in the degradation of cellular proteins. This finding was supported by a decrease in *β*-actin levels. Interestingly, co-exposure with monoHER suppressed p53 accumulation. A small increase in p53 levels may be observed after 24 h treatment with monoHER alone, which was not detected after 48 h. This increase was not seen in the other cells tested (see below). Although we have no clear explanation for this small increase it may reflect a more ROS-sensitive regulation of p53 in untreated HUVECs than in the other cells.

The pro-apoptotic Bcl2 family member Bax was constitutively expressed in untreated *HUVECs* and a shorter form of Bax of approximately 18 kDa appeared after treatment with DOX probably representing a cleaved or truncated form. The occurrence of this shorter variant was, however, prevented by monoHER. As predicted by the z-VAD-fmk sensitivity of DOX-induced apoptosis in these cells, robust caspase-9 and -3 cleavage was seen accompanied by processing of the caspase substrate PARP. MonoHER suppresses caspase activation. As controls, DMSO and monoHER alone did not trigger caspase activation.

In NeRCaMs, p53 expression was only detectable after DOX treatment and monoHER appeared to influence the post-translational modification of p53 as indicated by changes in the mobility of the p53 specific bands (Figure 3B). Doxorubicin (DOX) potently triggered caspase-9 activation as indicated by the strong decrease or even absence of the pro-form band at 24 and 48 h after treatment. Also strong PARP cleavage was observed. In light of the observed lack of effect of z-VAD-fmk treatment on DOX-induced cell death this may suggest that a caspase-independent form of cell death can be activated in parallel in these cells. Regardless of this, monoHER very effectively suppressed caspase-9 activation and PARP cleavage. We were not able to detect Bax or caspase-3 expression in NeRCaMs as the available antibodies did not cross-react with the rat counterparts.

Next, the ovarian cancer cell lines were investigated. A2780 cells (wild-type p53) showed clear DOX-induced p53 accumulation and PARP cleavage that could be suppressed by monoHER (Figure 3C). However, the mechanism of DOX-induced caspase activation in A2780 cells was different from that in HUVECs, with no or hardly any Bax, caspase-9 and -3 cleavage/activation, suggesting no primary role for the mitochondrial pathway in mediating DOX-dependent apoptosis in these cells. Nonetheless, A2780 cells displayed clear PARP cleavage after DOX exposure. In contrast, OVCAR-3 cells showed little or no evidence for DOX-induced p53, Bax or caspase activation or PARP cleavage (Figure 3D). This was not unexpected because these cells contain mutant p53 and show resistance against apoptosis.

## DISCUSSION

The antitumour effects of DOX results from preventing DNA replication and repair leading to cell cycle arrest and apoptosis (Capranico *et al*, 1990; Gewirtz, 1991; Binaschi *et al*, 1997). Doxorubicin (DOX)-induced cardiotoxicity was related to the activation of apoptosis in cardiomyocytes and endothelial cells (Wu *et al*, 2002; Yamanaka *et al*, 2003; Spallarossa *et al*, 2004). In these cells the main effects of DOX were mediated via the production of ROS, known for its potent cell damaging effects. Thus DOX-induced apoptosis, although via different routes, is a common mechanism in normal and cancer cells.

Interestingly, the potent ROS scavenger monoHER, which is presently in a clinical phase II study, showed a strong protection against the cardiotoxic effects of DOX without modulating its antitumour effects both *in vivo* and *in vitro* (van Acker *et al*, 1997; van Acker *et al*, 2000; Abou El Hassan *et al*, 2003a, 2003d). The anti-apoptotic role played by monoHER and the contribution to its selective protection is not studied yet. The present study was therefore aimed to compare the protective effects of monoHER against DOX-induced apoptosis in normal and cancer cells to unravel the mechanism of its selective protection.

Doxorubicin (DOX) induced concentration-dependent apoptosis both in HUVECs and NeRCaMs, which is in line with an earlier study (Wu *et al*, 2002). A2780 cells were also sensitive to induction of apoptosis by DOX, whereas OVCAR-3 cells were highly resistant. This resistance could be attributed to the presence of mutant p53, but may also be caused by other yet unknown apoptotic blockades. Employing the broad caspase inhibitor z-VAD-fmk revealed that DOX triggered caspase-dependent apoptosis in HUVECs and A2780 cells, and caspase-independent cell death in NeRCaMs. Combination treatment with monoHER was effective in suppressing both caspase-dependent (HUVECs and A2780) and -independent apoptosis (NeRCaMs). Assessment of the fold anti-apoptotic protection achieved showed that HUVECs and NeRCaMs were stronger protected by monoHER than A2780 cells. Examination of the molecular mechanisms that underlie the protective effects of monoHER indicated that it can strongly reduce the activation of DOX-induced p53 accumulation in cardiomyocytes, endothelial cells and A2780 cells, which contain wild-type p53 (Camarda *et al*, 2002), whereas as expected, no p53 response was detected in OVCAR-3 cells that express mutant p53. It is conceivable that the radical scavenging properties of monoHER cause the reduction of p53 accumulation, which is a known sensor of ROS-dependent toxicity (Uberti *et al*, 1999), leaving only topoisomerase 2-DNA damage as a trigger for p53 activation.

The suppressive effect of monoHER on the activation of caspase-9 and -3 and the substrate PARP can also be explained by the neutralisation of ROS-dependent triggers of caspase activation. Evidence for mitochondria-dependent activation of caspases was only obtained in HUVECs, in which DOX exposure resulted in the occurrence of a shorter form of BAX, which was suppressed by monoHER. This shorter form may be produced by cleavage of full-length Bax or may represent an alternatively spliced variant with mitochondria destabilising activity leading to the apoptosome-dependent activation of caspase-9 and the subsequent activation of apoptotic cell death (Youn *et al*, 2005). In A2780 cells the mitochondrial pathway seemed to be less involved in DOX-induced apoptosis because DOX did not show clear changes in BAX or pro-caspase-9 or -3 levels. However, PARP cleavage was clearly evident in A2780 cells and together with the fact that zVAD-fmk strongly suppressed DOX-induced apoptosis, we postulate that other yet unconfirmed mechanisms of caspase-dependent apoptosis are activated, such as the extrinsic pathway and caspase-8. The opposite holds true for NeRCaMs in which DOX activated p53 as shown by caspase-9 and PARP cleavage, whereas the addition of z-VAD-fmk only partially suppressed apoptosis indicative of caspase-independent cell death. In NeRCaMs, the observed caspase-9 activation seems to be merely a co-phenomenon of DOX-induced apoptosis. In line with that, Youn *et al* (2005) reported that DOX-induced death of the rat cardiac muscle cell line H9c2 was associated with p53 upregulation and caspase-independency. Altogether, this indicates the involvement of caspase-independent apoptotic routes in mediating death in cardiac myocytes by DOX. This cannot be generalised because in other cell types (in our study and in previous reports) caspases seem to play a role in DOX-induced cell death (Nakamura *et al*, 2000; Grassilli *et al*, 2004; Kalivendi *et al*, 2005). Together this supports the idea that more than one mechanism can mediate programmed cell death, which depends on both the death stimulus provided and the cell type studied (Bröker *et al*, 2005). MonoHER appears to protect against these diverse DOX-induced cell death signals, possibly because of the ability of monoHER to neutralise ROS. OVCAR-3 cells were different in their response to DOX compared to the other cell types. They showed hardly any apoptosis within the concentration range of 0.5–10 *μ*M, which is comparable to clinically achieved concentrations of DOX (0.3–5 *μ*M) (Gerwitz, 1999). MonoHER did cause cell cycle delay in G2/M in these cells but did not affect the small amount of DOX-induced cell death.

The finding that monoHER not only suppresses apoptosis in endothelial cells and cardiomyocytes at clinically relevant concentrations of DOX, but also in ovarian cancer cells raises the question whether monoHER may reduce the antitumour effects of DOX in the clinic. It should be noted that in the present study a concentration of 1 mM monoHER is used. This concentration is about threefold or sevenfold higher than the maximal peak plasma concentrations of monoHER found in the clinic (360 *μ*M) during the phase I study (Willems *et al*, 2006) or in mice (131 *μ*M) under protecting conditions (Abou El Hassan *et al*, 2003c). Thus, monoHER provides a potent protective effect against various routes of doxorubicin-induced cell death allowing an efficient protection of heart and endothelial cells reported previously as major targets in doxorubicin-induced cardiotoxicity. Importantly, the present study addresses the necessity not to raise the dose of monoHER above the presently used 1500 mg m^−2^ to avoid apoptosis-protecting concentrations which might adversely affect the antitumour activity of DOX.

Finally, we postulate that the tendency of monoHER to protect normal cells more than cancer cells may be attributed to the inherent proliferation capacity of cancer cells, even in confluent cultures as used in this study where the growth of tumour cells is less affected by contact inhibition. It can be envisioned that in slow- or non-proliferating cells, such as normal cardiomyocytes and endothelial cells, monoHER predominantly acts against the apoptotic effects of DOX and consequently offers more protection. This is supported by our results showing restricted monoHER protection of confluent but not proliferating endothelial cells after DOX treatment *in vitro* (Abou El Hassan *et al*, 2003d). On the other hand, the different effects of monoHER in terms of apoptosis suppression may reflect the activity of the intrinsic ROS defence systems present in cells, which may also be associated with different mechanisms of cell death activation. In this respect, multiple antioxidant defence systems/enzymes (for example, glutathione, thioredoxin and SOD) are active to varying degrees in different cell types thereby forming complex ROS-neutralising networks that are related to apoptosis (Haddad, 2004). In addition, cardiomyocytes were reported to have intrinsically low levels of antioxidant enzymes that may explain the potent protective function of monoHER in these cells (Hrdina *et al*, 2000). Also high activity of the MRP1/ GS-X pump in these cells was reported (Krause *et al*, 2007), which may export DOX as glutathione-S-conjugate out of the cells, thereby modifying its cytotoxic effects. However, previously we found that monoHER did not affect DOX levels in cardiac cells in mice or influence the biodistribution of DOX (Abou El Hassan *et al*, 2003b). Thus, although the level of ROS defense is likely to at least partially determine the protective effect of monoHER in cells, the high complexity of these defense mechanisms makes it difficult to discriminate between their relative contribution to monoHER protection and the relevance for counteracting DOX-induced apoptosis, and will require a systematic, more detailed analysis.

## Figures and Tables

**Table 1:** IC_50_ values (*μ*M) for endothelial (HUVECs), cardiac cells (NeRCaMs), OVCAR-3 and A2780 cells

**Cell type**	**DOX**	**DOX+MH**	**Fold protection**
HUVECs	0.75	10	13
NeRCaMs	0.5	7.5	15
A2780	1.5	8	5.3
OVCAR-3	>10	>10	—
